# Investigating the Protein Signature of Adamantinomatous Craniopharyngioma Pediatric Brain Tumor Tissue: Towards the Comprehension of Its Aggressive Behavior

**DOI:** 10.1155/2019/3609789

**Published:** 2019-05-02

**Authors:** Claudia Martelli, Riccardo Serra, Ilaria Inserra, Diana Valeria Rossetti, Federica Iavarone, Federica Vincenzoni, Massimo Castagnola, Andrea Urbani, Gianpiero Tamburrini, Massimo Caldarelli, Luca Massimi, Claudia Desiderio

**Affiliations:** ^1^Istituto di Biochimica e Biochimica Clinica, Università Cattolica del Sacro Cuore, Roma, Italy; ^2^Università Cattolica del Sacro Cuore, Istituto di Neurochirurgia, Roma, Italy; ^3^Fondazione Policlinico Universitario A. Gemelli IRCCS, Roma, Italy; ^4^Istituto di Chimica del Riconoscimento Molecolare, Consiglio Nazionale delle Ricerche, Roma, Italy; ^5^Laboratorio di Proteomica e Metabonomica, IRCCS-Fondazione Santa Lucia, Roma, Italy; ^6^Area Diagnostica di Laboratorio, Fondazione Policlinico Universitario Agostino Gemelli-IRCCS, Roma, Italy; ^7^Fondazione Policlinico Universitario A. Gemelli IRCCS, Neurochirurgia Pediatrica, Roma, Italy

## Abstract

Although histologically benign, adamantinomatous craniopharyngioma (AC) pediatric brain tumor is a locally aggressive disease that frequently determines symptoms and hormonal dysfunctions related to the mass effect on the surrounding structures. Another typical feature of this benign neoplasm is the presence of voluminous liquid cysts frequently associated with the solid component. Even if studies have been devoted to the proteomic characterization of the tumor intracystic fluid, poor explorations have been performed on its solid part, principally investigated by transcriptomics technologies. In the present study, seven specimens of AC whole tumor tissue have been analyzed by LC-MS for a preliminary assessment of the proteomic profile by a top-down/bottom-up integrated approach. Thymosin beta 4, ubiquitin, calmodulin, S100 proteins, prothymosin *α* isoform 2, alpha-defensins 1-4, and fragments largely belonging to vimentin, hemoglobin, and glial fibrillary acidic protein characterized the intact proteome. The identification of alpha-defensins, formerly characterized in AC intracystic fluid, reinforces the hypothesis of a role for inflammation in tumor pathogenesis. A total number of 1798 unique elements were identified by a bottom-up approach with a special focus on the 433 proteins commonly characterized in the 85.7% of the samples analyzed. Their gene ontology classification evidenced the involvement of the adherence system, intermediate filaments, and actin cytoskeleton in tumor pathogenesis and of elements part of the Wnt, FGF, and EGFR signaling pathways. In addition, proteins involved in calcium modulation, innate immunity, inflammation, CCKR and integrin signaling, and gonadotropin-releasing hormone receptor pathways were also outlined. Further than confirming proteomic data previously obtained on AC intracystic fluid, these results offer a preliminary overview of the AC whole tissue protein phenotype, adding new hints towards the comprehension of this still obscure pediatric brain tumor.

## 1. Introduction

Adamantinomatous craniopharyngioma (AC) is the most common sellar tumor in the pediatric age representing 5-11% of intracranial tumors with an incidence of 1.53-2.92/100000 per year under 15 years [1, 2]. Owing to the aggressive behavior of the adamantinomatous variant, this benign neoplasm tends to infiltrate the adjacent eloquent regions, the optic pathways, the Willis' circle, and the hypothalamus, setting up a typical pattern of chronic recurrence that can last for years and that represents one of the main pathologic features of this tumor. According to recent data, the rate of recurrence of craniopharyngiomas is higher in children than in adults and can be as high as 60% after radical or subtotal resection [3]. Recent studies, based on genetic approaches and immunohistochemical/ELISA analysis on AC tissues and *in vitro* and *in vivo* models, provided a relevant contribution to the understanding of the molecular pathways and gene alterations involved in tumor onset and progression. Moreover, they further clarify the role of the Wnt pathway and the upregulation of the EGFR pathway, SHH signaling, and specific matrix metallopeptidases, as recently reviewed [4–7]. Recent evidence highlighted the pathogenic role of Wnt/beta-catenin in AC after the discovery of a small population of stem cells responsible for its growth and proliferation and of a number of associated beta-catenin mutations [8, 9]. Several studies were devoted to disclose distinct molecular profiles for AC with respect to the papillary histotype. Other characteristic features displayed in AC are the overexpression of therapeutic target genes of the EGFR/ERBB pathway, including AREG, EGFR, and ERBB3, of SHH signaling, including the SHH 19 kDa active form, of the Wnt pathway, with 32-fold enrichment of beta-catenin/LEF/TCF target genes and the abnormal expression of LEF1 and WNT5A [10]. In addition, overexpression of diverse isoforms of matrix metalloproteinases MMP9 and MMP12, MAP2, tenascin C (TNC), and stem cell marker CD133 were found, while CD44 and claudin-1 resulted to be downregulated [10]. In the same study, the gene ontology enrichment of the AC gene signature classified the majority of them as involved in odontogenic (DLX2, ODAM, AMBN, AMELX, ENAM, TP63, EDAR, SHH, and FGF4), epidermal (including several keratins, KRT5 13-16, 31, 34, and 85, and laminins LAMA3 and LAMC2), and epithelial development. Together with the tumor stem cell markers, CD44 and CD133, AC were found also to express the paracrine factors, BMP4, FGF, and SHH [5]. The downregulation of cell adhesion molecule claudin-1 distinguished AC from other craniopharyngioma subtypes and from the Rathke's cleft benign cysts (RCC) [11]. On the opposite, the expression of epithelial cell adhesion molecule EpCAM [12] and fascin-1 in the beta-catenin-accumulating cells [5] was found. A transcriptional study on recurrent AC disclosed the upregulation of 16 genes and a significant association of tumor relapse with the expression of CXCL12 and CXCR4 [13]. A proteomic investigation by two-dimensional gel electrophoresis disclosed a high level of annexin A2 (ANXA2) in AC with respect to normal brain tissue and ascribed to the protein a potential role of a prognostic biomarker for possible application in the patient follow-up [14]. A very recent comprehensive transcriptomic study on the human AC solid component originally mapped the differential gene expression profiles associated with diverse cell types by using laser capture microdissection. The transcriptome of the tumor epithelium, including beta-catenin-accumulating cluster cells and palisading epithelium, and the glial reactive tissue compartments was indeed investigated [15]. Gene enrichment analysis associated the gene expression signature of inflammatory response to glial reactive tissue and that of Wnt signaling to the tumor tissue, the latter stronger in cell clusters compared to palisading epithelial cells. This study evidenced the overexpression in AC of genes related to odontogenesis, such as ameloblast transcription factors (BCL11B, MSX2), to enamel (ENAM, AMELX, AMELY, AMBN), and to proteinase (MMP20, KLK4) genes. In particular, enrichment of the gene signature of the enamel knot was associated with the cluster cells, also confirmed by immunofluorescence of the ectodysplasin receptor (EDAR). Moreover, a strong molecular similarity between the palisading epithelium and enamel epithelium was recognized. Cluster cells were also investigated for their paracrine signaling activity by RNA profiling, showing overexpression, with respect to the palisading epithelium and reactive glia, of multiple ligands and secreted factors of FGF, BMP, Wnt, SHH, MAPK/ERK, and TGF*β* pathways, responsible for the activation of this signaling in the neighboring cells. On this basis, preclinical studies *in vitro* on human and mouse AC of the MAPK/ERK pathway inhibitor drug trametinib resulted to be promising for potential AC treatment [15]. Evidence of immune-related gene expression inside the major cellular patterns of AC was provided through transcriptomic analysis of whole tissues, and furthermore, cytokine levels were evaluated by ELISA. Furthermore, the presence of an immune infiltrate and of other markers of inflammation was evaluated by means of LC-MS proteome analysis on the intracystic fluid protein digest, revealing an enrichment of proteins involved in immune/defense response, inflammation, and steroid metabolism. ELISA analysis of cytokines showed activation of the inflammasome complex in AC, with the probable involvement of cholesterol.

Our previous proteomic studies by top-down and bottom-up LC-MS platforms on AC intracystic fluid [16–19] were able to evidence the presence of alpha-defensins and beta-thymosin peptides suggesting that inflammation is involved, at least partly, in AC pathogenesis and cyst development. This hypothesis was confirmed by Donson et al. [20]. The study, carried out by cytometric and transcriptomic analyses, found high levels of inflammatory cytokines, chemokines, and immunosuppressive factors on AC tissue and intracystic fluid in comparison to intracystic fluid and tissues of other brain tumors and to normal tissue.

Following our previous investigations on AC intracystic fluid proteome, this study is aimed at preliminarily exploring the protein profile of the AC solid component by nano-LC-Orbitrap Elite-MS analysis of whole tissue homogenates utilizing an integrated top-down/bottom-up platform.

## 2. Material and Methods

### 2.1. Chemicals

All organic solvents were of LC-MS grade. Iodoacetamide (IAA), DL-dithiothreitol (DTT), Trizma® hydrochloride, sodium deoxycholate, urea powder, sodium dodecyl sulfate (SDS), Tergitol-type NP-40, and acetone were from Sigma-Aldrich (St. Louis, Missouri, USA). TFA and sodium chloride were from Mallinckrodt Baker B.V. (Deventer, The Netherlands) and Fluka (Sigma-Aldrich Chemie GmbH, Buchs, Switzerland), respectively. Acetonitrile (ACN), methanol (MeOH), EDTA, and formic acid (FA) were from Merck (Darmstadt, Germany), while ethanol (EtOH) absolute and water were from Prolabo (Fontenay-sous-Bois, France). Trypsin (Gold MS Grade) was from Promega (Madison, Wisconsin, USA), and Halt™ Protease and Phosphatase Inhibitor Cocktail (100x) were from Thermo Fisher Scientific (Rockford, IL).

### 2.2. Instrumentation

Tissue sample homogenization and sonication were carried out by means of the Wheaton® 903475 Overhead Stirrer apparatus (Wheaton, Millville, New Jersey, USA) and Branson Sonifier 450 (Branson Ultrasonics, Danbury, USA), respectively. Total protein concentration was determined in duplicate by Bradford assay (Bio-Rad Laboratories, Hercules, California, USA) by means of a UV-Vis spectrophotometer (8453 UV-Vis Supplies, Agilent Technologies, Waldbronn, Germany) using BSA as the protein of reference. HPLC-ESI-MS/MS analyses were performed on an UltiMate 3000 RSLCnano System coupled to an Orbitrap Elite MS detector with EASY-Spray nanoESI source (Thermo Fisher Scientific). EASY-Spray columns 15 cm × 50 *μ*m ID, PepMap C18 (2 *μ*m particles, 100 Å pore size), and 15 cm × 75 *μ*m ID, PepMap C18 (5 *μ*m particles, 300 Å pore size) (Thermo Fisher Scientific), were used for bottom-up and top-down analyses, respectively, in coupling to an Acclaim PepMap 100 cartridge (C18, 5 *μ*m, 100 Å, 300 *μ*m i.d. × 5 mm) (Thermo Fisher Scientific).

### 2.3. AC Tissue Sample Collection and Treatment

Bioptic tissues were obtained from 7 patients (5 males, 2 females, 8-18 years, mean age 10.7 years) affected by AC in the suprasellar or sellar/suprasellar region with associated cyst who underwent surgical removal of the tumor at our Institution. The study was realized under the approval of the local Ethical Committee. Samples were collected under sterile conditions during surgery and immediately stored at -80°C. Tissue samples were thawed on ice, washed with cold PBS containing protease and phosphatase inhibitor cocktail to remove blood contamination, and weighed. Samples were then homogenized with a tissue grinder for 3 min in a RIPA buffer (Tris-HCl 50 mM pH 8), NaCl 150 mM, sodium deoxycholate 0.5% (*v*/*v*), SDS 0.1% (*v*/*v*), NP-40 1% (*v*/*v*), and EDTA (1 mM volume containing 1% (*v*/*v*) protease inhibitor cocktail) to obtain a final tissue/buffer homogenate concentration of 30 *μ*g/*μ*L. After storage on ice for 30 min, with a brief stirring every few minutes, the homogenates were sonicated (2 min in ice at 180 watts) in rounds of alternate 10 sec sonication/rest and centrifuged (10000 g × 10 min, +4°C) and the resulting supernatants collected.

For bottom-up analyses, a volume of supernatant, equal to 500 *μ*g of total protein content, was added with 6x volume of cold EtOH : MeOH : acetone : water (49 : 24.5 : 24.5 : 2, *v*/*v*), stirred, and stored overnight at -80°C for protein precipitation. After centrifugation (30 min × 23800 g, +4°C), the pellet was recovered and underwent *n* = 4 repeated steps of protein precipitation in 1 mL of cold water : acetone (20 : 80, *v*/*v*) followed by 30 min storage at -80°C and centrifugation (23800 g, +4°C), each time discarding the supernatants and recovering the protein precipitate. The pellet was evaporated to dryness and suspended in 100 *μ*L of urea buffer (6 M urea, 100 mM Tris-HCl pH 7.8) through sonication (3 × 10 sec). After centrifugation (22800 g × 10 min), the total protein content was determined by Bradford assay. 416 *μ*g of total proteins for each sample was treated for disulfide bond reduction with 10 mM DTT for 1 h at +37°C and alkylated with 20 mM IAA at +37°C for 1 h in the dark. IAA excess was removed by incubation of the sample with 1.61 mM DTT at +37°C for 20 min. Sample digestion was carried out overnight at +37°C using trypsin in 1 : 50 (*w*/*w*) ratio with respect to the protein content. Enzymatic digestion was stopped by addition of 0.1% FA (*v*/*v*). The resulting peptides underwent a clean-up step using C18 ZipTip pipette tips (Millipore Corporation; Billerica, Massachusetts, USA).

For top-down analysis, a supernatant volume equal to 500 *μ*g total protein content was added with 4x volume of cold (-20°C) acetone, vortex-mixed, and incubated for 60 min at -20°C. After centrifugation (10 min × 14000 g, +4°C), the supernatant was discarded. The pellet underwent an additional precipitation cycle. The resulting pellets were evaporated to dryness at room temperature for 30 min avoiding to overdry and then suspended in 0.4% (*v*/*v*) aqueous TFA. The total protein content was quantified by Bradford assay.

### 2.4. Nano-HPLC-nanoESI-Orbitrap Elite Analysis

Bottom-up nanoHPLC-MS/MS analyses were performed using aqueous solution of FA (0.1%, *v*/*v*) as eluent A and ACN/FA (99.9 : 0.1, *v*/*v*) as eluent B in the following gradient elution: (i) 5% B (2 min), (ii) from 5% to 60% B (120 min), (iii) from 60% B to 99% (15 min), (iv) 99% B (10 min), (v) from 99% to 5% B (2 min), and (vi) 5% B (13 min) at a flow rate of 0.3 *μ*L/min. The injection volume was 5 *μ*L corresponding to 1 *μ*g of total protein content per sample. Peptide trapping and concentration were obtained loading the sample for 5 min into the Acclaim PepMap 100 nano-trap cartridge operating at 10 *μ*L/min in eluent A. Chromatographic separations were performed at 40°C. The Orbitrap Elite instrument was operating in positive ionization mode, performing MS/MS fragmentation by collision-induced dissociation (CID, 35% normalized collision energy) of the 20 most intense signals of each spectrum, measured at a 60000 resolution in 350-2000 *m*/*z* acquisition range, in data-dependent scan (DDS) mode (activation time of 10 ms). The minimum signal was set to 500.0, the isolation width to 2 *m*/*z*, the default charge state to +2, and the activation *Q* to 0.25. MS/MS spectra acquisition was performed in the linear ion trap at a normal scan rate.

### 2.5. Data Analysis

Top-down data were elaborated by means of Xcalibur (version 2.0.7 SP1, Thermo Fisher Scientific) and its deconvolution tool by manual inspection of tandem MS spectra, matching the experimental/theoretical results by using ExPASy UniProtKb database and proteomics tools (https://www.expasy.org/tools/) and Proteome Discoverer 1.4 software (version 1.4.1.14, Thermo Fisher Scientific). Relative quantitation was assessed by comparing the protein/peptide peak area (signal/noise ratio > 5) obtained in the extracted ion current (XIC) plot by isolation of the ion current signals of the relative multiply charged ions (*m*/*z*) from the total ion current (TIC) profile. The peak area values were normalized to the total protein content of the relative sample analyzed and used for a comparative evaluation.

MS and MS/MS data obtained from bottom-up analyses were elaborated by Proteome Discoverer 1.4 software (version 1.4.1.14, Thermo Fisher Scientific), based on the SEQUEST HT cluster as search engine against the Swiss-Prot Homo Sapiens proteome (UniProtKb, Swiss-Prot, Homo Sapiens, released on March 2018) and setting the following parameters: minimum precursor mass 350 Da, maximum precursor mass 10000 Da, total intensity threshold 0.0, minimum peak count 1, signal-to-noise (S/N) threshold 1.5, precursor mass tolerance 10 ppm, fragment mass tolerance 0.5 Da, use average precursor mass False, and use average fragment mass False. The trypsin enzyme was set with a maximum of 2 missed cleavage sites. For data elaborations, the minimum and maximum peptide length was 6 and 144 residues, respectively. Dynamic methionine oxidation (+15.99 Da) and static carbamidomethylation of cysteine (+57.02 Da) were also set. Protein and peptide spectra matches were validated by the calculation of the false discovery rate (FDR) using the Percolator node. The strict target FDR value was set at 0.01, while the relaxed value was set at 0.05. Protein identification results were filtered for high peptide confidence: peptide rank 1, 2 peptides per protein, and peptide length ≥ 9 amino acids. For top-down data elaboration by Proteome Discoverer, the following result filters were applied: high peptide confidence and peptide rank 1.

Sample grouping was carried out utilizing the Venn Diagrams tool (http://bioinformatics.psb.ugent.be/webtools/Venn/). Gene Ontology (GO) analysis and pathway classification of the identified proteins were performed by Protein ANalysis THrough Evolutionary Relationships (PANTHER) Classification System (version 11.0) [21]. Pathway overrepresentation analysis was performed PANTHER and REACTOME [22] databases.

## 3. Results

LC-MS top-down and bottom-up proteomic analysis was performed on whole AC tissues to characterize the proteome in either its entire or its digested form and to disclose common proteomic signatures. On the one hand, the top-down approach revealed small proteins and peptides, allowing the identification of naturally occurring protein fragmentome, proteoforms, and PTMs. On the other hand, the bottom-up strategy, including the identification of proteins of high molecular weight, produced a large protein data set on which was based the gene ontology analysis and pathway classification.

### 3.1. Top-Down Approach Protein Identification

The LC-MS data obtained by the analysis of the undigested protein extracts of AC tissue homogenates have been elaborated by both Proteome Discoverer 1.4 software and manual inspection of the MS and MS/MS spectra recorded along the chromatogram. The results of LC-MS analysis software elaboration and the manual identifications of tandem MS spectra are reported in Supplementary File S1 and S2, respectively.  Table 1 lists the proteins and peptides characterized in at least four out of the total seven AC tissues analyzed. The protein and peptide elements commonly identified in at least 6/7 samples, typifying the AC tissue, are marked in bold. In accordance with the features of the top-down proteomic platform, the list includes elements with molecular weight ranging from 1 to 17 kDa, carrying diverse PTMs mainly represented by N-terminal acetylation and C-terminal truncations. In addition to the identification of full-chain sequences of proteins and peptides, including the isoform 2 of prothymosin alpha, calmodulin 1, S100 proteins, ubiquitin, alpha-defensins, and beta-thymosin peptides, several protein fragments have been also identified. The latter were for greater part fragments of glial fibrillary acidic protein, vimentin, and hemoglobin globin chains. Furthermore, fragments of the fibrinogen alpha chain, alpha-1-antichymotrypsin, alpha-1-antitrypsin, brain acid-soluble protein 1, aquaporin-4, serum albumin, histones H1 and H2B, tubulin alpha chain, neuromodulin, and microtubule-associated protein 1B were characterized. C-terminal truncation generates the *des-IS* proteoform of thymosin beta 10 and the *des-GG* form of ubiquitin. Both these proteoforms, previously identified in medulloblastoma pediatric brain tumor tissue [23], were identified in the AC solid component. Differently from medulloblastoma, in AC tissue no C-terminal truncated proteoforms of thymosin beta 4 were observed, while its oxidized form and fragments were detected. In addition to beta-thymosins, also alpha-thymosins have been characterized in AC, including prothymosin alpha isoform 2 and the prothymosin alpha bioactive peptide fragments named thymosin alpha 1 and alpha 11. Alike *des-IS* thymosin beta 10, these fragments are typical markers of malignant medulloblastoma [23]. Although excluded from the list because of being recognized in a few samples, S100A6 was also detected in its uncysteinylated (10085.31 Da) and glutathionylated (10390.37 Da) forms (data not shown), the latter already described in the literature [24].

It is noteworthy to underline that 67% of the proteins were identified by both proteomic approaches, although with different contributions to the understanding of AC tumor proteome: while the bottom-up strategy ascertained protein presence through the identification of proteotypic peptides, the top-down approach, by looking at their intact forms, precisely recognized proteoforms and PTMs and identifies naturally occurring protein fragments. The latter, called cryptides [25], can be modulated under pathologic conditions and can exert a biological activity different from that of the parent protein. Examples include the bioactive fibrinopeptide A (1536.69 Da) corresponding to fragments 20-35 of the fibrinogen alpha chain and the C-terminal fragments 384-418 (4046.20 Da) of alpha-1-antitrypsin, enclosed in the C-terminal peptide 375-418 with immunomodulatory activity [26]. It is also noteworthy to underline, within the diverse hemoglobin chains' fragments, the characterization in 5/7 AC samples of fragment 33-42 of hemoglobin beta chain called LVV-hemorphin-7, a nonclassical opioid peptide identified in our previous paper in cerebrospinal fluid of posterior cranial fossa pediatric brain tumors with a potential role as a prognostic biomarker [27]. This peptide holds multiple biological activities including a role in the maintenance of homeostasis [28]. Recently, the expression of hemoglobin chains in neurons and glial cells, in endothelial and tumor vessels, and the potential role of hemoglobin and hemorphin peptides within brain tumors [29] shed a new light on the investigation of this nonepithelial protein and its fragment. The fragment 25-48 of serum albumin was detected in four samples, and given its frequent identification in biological fluids and tissues [25], this fragment could have a biological significance.

The proteins exclusively identified by the top-down strategy include ubiquitin, thymosin beta 10, some histone fragments, and alpha-defensin peptides. Regarding alpha-defensins, only by means of the top-down strategy was it possible to distinguish alpha-defensin 1 and alpha-defensin 2 peptides, both corresponding to different sequence traits (65-94 and 66-94) of the neutrophil defensin 1 chain. Similarly, the isoform 2 of prothymosin alpha was identified only in the undigested proteome analysis, where the molecular mass of the entire protein and the MS/MS sequencing allowed characterizing the absence of the Glu_40_ residue in the sequence.

### 3.2. Protein Identification by Bottom-Up Platform

**Figure 1:**
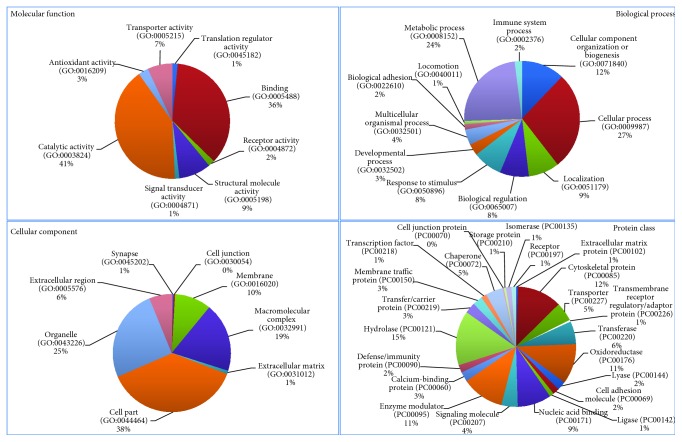
Molecular function, biological process, cellular component, and protein class PANTHER database Gene Ontology (GO) classification of the proteins characterized by bottom-up strategy in at least six AC tissue specimens.

**Figure 2:**
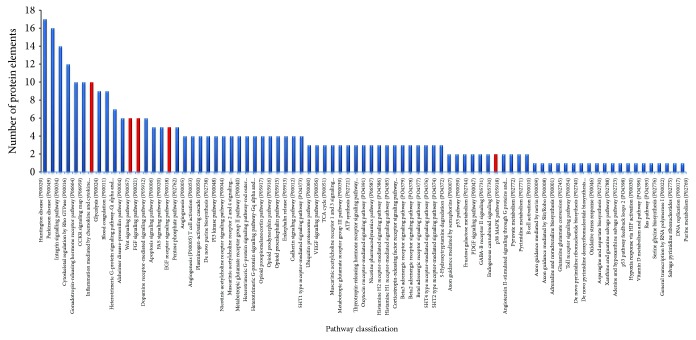
PANTHER pathway classification of the protein elements identified in at least six AC whole tissues analyzed by bottom-up proteomic analysis. Red color evidences the inflammation mediated by chemokine and cytokine, Wnt, FGF, EGF receptor, and p38MAPK pathways.

**Figure 3:**
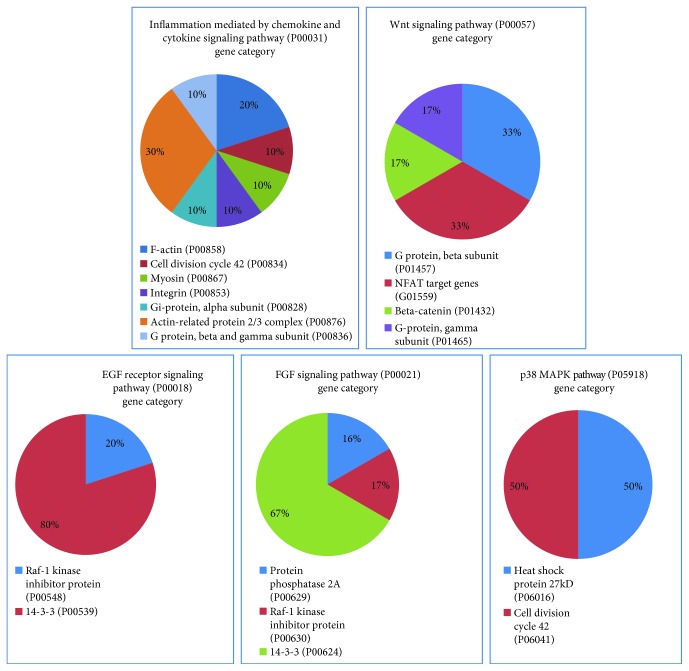
Gene category distribution of the protein elements classified by PANTHER GO analysis in inflammation mediated by chemokine and cytokine, Wnt, FGF, EGF receptor, and p23 MAPK pathways.

Bottom-up LC-MS data analysis has been elaborated by Proteome Discoverer 1.4 software based on the SEQUEST HT cluster as a search engine and using the Percolator node based on false discovery rate (FDR) calculation for validation of protein and peptide spectra matches. The Proteome Discoverer multireport data file of bottom-up identifications in the AC tissue specimens analyzed is reported in Supplementary Data File S3. Venn diagram elaboration of the protein identifications per single specimens revealed a number of 1798 total unique elements, of which 205 were shared by all the seven samples analyzed and 228 were found in at least six out of them (Supplementary File Table S1). These 433 proteins were explored for gene ontology (GO) analysis and pathway classification by the Protein ANalysis THrough Evolutionary Relationships (PANTHER) Classification System (version 9.0). As shown in the pie chart of Figure 1, the majority of the 433 proteins were cataloged as components of the organelle (25%), cell part (38%), and macromolecular complex (19%) and mainly classified in catalytic (41%) and binding (36%) activities and cellular (27%) and metabolic (24%) biological processes. Of them, 15% and 12% belong to hydrolase and cytoskeletal protein classes, respectively, and 11% are oxidoreductase and enzyme modulators. The GO analysis by the PANTHER tool relative to human pathways classified the identified proteins in numerous pathways.  Figure 2 shows the classified pathways in function of the number of protein elements characterized. Although the histological complexity of the AC solid component makes difficult to decipher this classification, it is noteworthy to underline that among the most represented pathways, those related to Wnt, FGF, EGFR, and inflammatory signaling (marked in red color) were uniformly identified, in accordance with previous findings [4, 5]. Considering the role of the MAPK/ERK pathway in AC and the promising results obtained in a preclinical study with the MAPK/ERK inhibitor trametinib [15], it is important to highlight the presence of a number of elements that are part of the p38MAPK pathway, which could direct future *in vitro* testing of selective inhibitors. The gene categories included in the Wnt, FGF, EGFR, inflammation, and p38MAPK pathways are illustrated in the pie chart of Figure 3. The input gene list for pathway classification, the PANTHER pathway classification, and their relative gene component lists are reported in Supplementary File S4–S6, respectively. In addition to inflammation, Wnt, FGF, and EGFR signaling pathways, the pathways related to neurodegenerative diseases, integrin signaling, cytoskeletal regulation by Rho GTPase, gonadotropin-releasing hormone receptor, CCKR signaling, glycolysis, and blood coagulation were among the most highly represented.

Another interesting link is that between the cholecystokinin receptor (CCKR) and gonadotropin-releasing hormone receptor pathways, both interconnected to the beta-catenin, p38MAPK, and other pathways expressed in AC [30–32]. A literature-based reconstruction of the CCKR signaling map revealed a complex network of molecular interactions, which suggested a new hypothesis on the mechanisms regulating cellular proliferation, migration, and resistance to apoptosis. This network includes in fact members of the EGFR, MAPK, and gonadotropin-releasing hormone receptor pathways, with downstream suppression of caspases and activation of clusterin expression [31]. In addition, cholecystokinin and its transduction pathway were also reported to exert an anti-inflammatory effect through p38MAPK signaling activation in rats with lipopolysaccharide-induced endotoxic shock [30]. Considering the importance of inflammation in AC pathogenesis, these findings could suggest further investigations of CCKR involvement in the tumor.

The complete list of proteins identified in at least 6 out of 7 samples by top-down and bottom-up integrated platforms has been further submitted to pathway overrepresentation analysis by PANTHER and REACTOME tools.

The analysis by PANTHER, using Fisher's exact test with FDR correction with respect to the human genome as reference, evidenced twelve pathways with FDR < 0.05 (list in Table 2; detailed results reported in Supplementary Data File S7). The REACTOME analysis tool, producing a hierarchic list of the pathway events associated with the protein UniProt accession list submitted, shows the results with a probability score, which is corrected for FDR using the Benjamini-Hochberg method (top 25 pathways' list in Table 3; detailed results reported in Supplementary Data File S8). The hemostasis and immune system and their hierarchical levels, namely, neutrophil degranulation, platelet degranulation, activation, signaling, and aggregation, and response to elevated platelet cytosolic Ca^2+^, are the most prominent pathways in the analyzed samples. Although not in the top 25 list, it is important to highlight the statistically significant overrepresentation of Wnt and beta-catenin/Wnt-independent signaling as well as MAPK and Hedgehog signaling (Supplementary File S8).

## 4. Discussion

Although genetic analysis identified specific pathway alterations and gene mutations associated with AC pathogenesis, the molecular mechanisms underlying disease onset remain to be fully clarified, together with the identification of effective targets of therapy. The integrated top-down/bottom-up LC-MS analysis provided a first overview of the whole tissue protein phenotype and confirmed previous findings mainly based on transcriptomic data.

Of relevance was the widespread identification in AC tissue of beta-catenin and beta-catenin-related proteins, uniformly present in all samples, suggesting the involvement of this pathway as a primary driver of AC tumorigenesis, with the possible contribution of intermediate filaments and actin cytoskeleton signaling. The role of beta-catenin has been studied for decades, and undoubtable evidence describes it as a pivotal player in colon cancer tumors and, more generally, in a series of epithelium-derived malignancies [33, 34]. Similarly, the involvement of beta-catenin in pituitary AC tumorigenesis has been known for several years and is also supported by the presence of mutations in its encoding gene in several cases [35, 36]. Moreover, recent studies have shed new light on the presumptive role of beta-catenin in AC pathogenesis, also considering the discussed presence of pituitary stem cells (PSCs) and their paracrine role on the surrounding cells [9]. In AC, hyperactivation of the Wnt/beta-catenin pathway was extensively demonstrated, together with evidence of beta-catenin intranuclear accumulation in a small number of cluster cells [36, 37] that express high levels of mitogenic signals and various chemokines and chemokine receptors. Furthermore, the upregulation of the beta-catenin pathway activates matrix metalloproteinase-7 (MMP-7) and c-Myc, responsible for promoting cell growth and invasion and progression of malignant tumors. Wnt/beta-catenin signaling, pivotal for pituitary development and hormone production, was also hyperactivated in a novel murine model of human AC: this tumor shares a number of traits (e.g., cyst formation, cell clusters with beta-catenin cytoplasmic accumulation, and Axin2-Lef1-Bmp4 expression) with its human counterpart, remarking once again the hypothesized role of this intracellular mediator in AC pathogenesis [38].

Beta-catenin-related elements have been identified in all AC samples also by the top-down approach, such as beta-thymosins peptides and S100A6 protein. Beta-thymosins, already described in the tumor intracystic fluid [17–19], could have a role in the cellular mechanisms driving the development and progression of this neoplasm. In colon cancer, the overexpression of thymosin beta 4 peptide is in fact associated with tumor invasion by promoting the downregulation of E-cadherin, the sequestering agent of beta-catenin [39], with the consequent loss of cell adhesion and activation of the beta-catenin signal. This information, together with the presented results, might suggest a close relationship between thymosin beta 4 and beta-catenin accumulation, in relation to AC tumor growth and invasion. The identification of S100A6 in AC samples was also consistent with previous findings [40], and its role in the ubiquitin-dependent degradation of beta-catenin highlights another possible link to this intracellular mediator [24]. In the AC tissue analyzed, S100A6 was usually identified in its cysteinylated form: this modification appears to regulate the biological function of S100A6 via redox modifications, influencing its calcium-binding capability, protein interactions, and the intracellular location in response to stress [24]. The S-thiolation of the S100A6 protein, only minimally detected in its unmodified form, could perhaps have a role in the disease by altering the beta-catenin pathway.

Besides S100A6, other S100 calcium-binding proteins, related to several diseases and with different intra- and extracellular functions [41], have been identified in AC by the top-down or the bottom-up platform, namely, S100A1, S100A2, S100A3, S100A8, S100A9, S100A10, S100A11, S100A13, S100A14, S100A16, and S100B. S100 proteins are involved in the regulation of cytoskeleton, intracellular calcium signaling, and cell proliferation via indirect p53 modulation and have therefore a role in cancer pathogenesis and inflammation [41]. Their characterization in AC tissue could be connected either to the aggressive behavior of the tumor or to the role of calcium in the disease, also supported by the identification of other related elements, such as calmodulin and the evidence of calcium flecks in the intracystic tumor fluid. Among the calcium-related proteins, it is noteworthy to remark the ubiquitous characterization in AC samples of ANXA2: following a comparative evaluation of its area values (Supplementary File S3), ANXA2 was found to be one of the most abundant proteins identified in AC samples. This result was in line with previous evidence ascribing to this protein a relevant prognostic value in AC disease [14], and it suggests the use of targeted LC-MS as a reliable method for its biomarker profiling.

The analysis of the undigested proteome provided a new perspective and better understanding of the naturally occurring fragmentome of AC tissue: of particular interest was the description of hemoglobin-derived peptides, C-terminal truncated forms of ubiquitin, and beta-thymosins peptides, all previously characterized in pediatric tumors of the posterior cranial fossa [23, 42] and of unclear biological significance in these diseases.

Alpha-defensins 1-4, a family of small neutrophil-derived peptides previously identified in the AC cystic fluid [16, 17], appears to have a central role in the innate immune response and inflammation, beside its well-known antimicrobial and antiviral properties. Their finding in the solid component of AC could confirm once again the role of inflammation in AC development, as also recently outlined by solid and cyst tumor component cytokine and chemokine expression profiles [20]. The anti-inflammatory mechanism of alpha-defensin 1, the most abundant peptide of the group, was recently attributed to its capability to modulate protein translation in macrophages [43]. Moreover, it is important to remark the detection in AC tissue of the fragments 387-423 and 390-423 of alpha-1-antichymotrypsin, the first previously identified in the tumor intracystic fluid [17] and involved in the host acute response to inflammation. These fragments have been reported as potential biomarkers of gliomas [44] and acute renal allograft rejection [45] and are possibly produced by cathepsin D protease activity [46].

**Figure 4:**
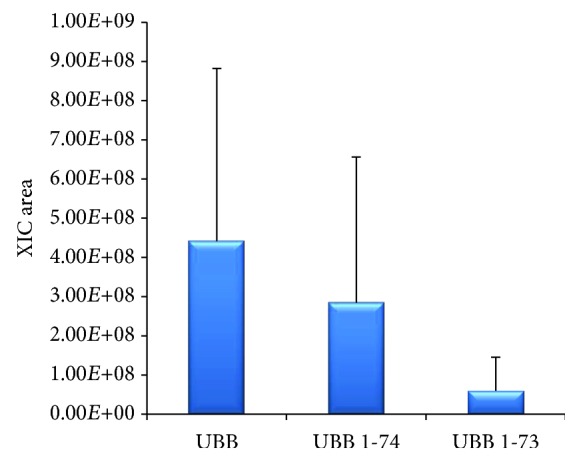
Relative quantitation of ubiquitin (UBB) and its des-GG (UBB 1-74) and des-RGG (UBB 1-73) C-terminal truncated forms in AC tissues.

The overrepresented pathway list resulting from PANTHER analysis interestingly includes Parkinson disease- (PD-) related signaling. Remarkably, a connection between PD and cancer supported by epidemiological and genetic evidences was recently outlined [47]. Chronic inflammation with the alteration of the COX2 enzyme and of the ubiquitin proteasome system seems to play a central role in this link, and in particular, the ubiquitin carboxyl-terminal hydrolase isozyme L1 (UCHL1) is among the genes involved in both PD and cancer pathogenesis. UCHL1 mutations produce alterations of the ubiquitin-dependent protein degradation system that cause protein misfolding and aggregation in neurodegenerative disorders [47]. Moreover, its aberrant expression was associated with cell invasion, transformation, and self-renewal capacity in high-grade pediatric gliomas [48]. Interestingly, both COX2 and UCHL1 are included among the most represented proteins in AC tissue, and UCHL1 was identified in the majority of the AC samples. The detection of the des-GG ubiquitin proteoform, already discussed in the top-down identification paragraph, could be the phenotypic expression of its activity, since the enzyme cleaves the C-terminal Gly bond of the ubiquitin monomeric chain. From relative quantitation data, the des-GG form accounts in AC tissue for levels similar to that of the entire monomer, also when accounting for the high interindividual variability (Figure 4).

The overrepresentation of the glycolysis pathway (about 24-fold enrichment) could be compatible with the Warburg effect typical of malignancies and associated with cell proliferation, while the activation of extra-mitochondrial glucose oxidation by the pentose phosphate pathway (about 30 fold enrichment), being a source of ribose-5-phosphate for purine and pyrimidine nucleotide production, could be related to the high rate of DNA replication and cell division [49]. These two pathways underwent a reciprocal metabolic switch associated with cell proliferation and migration in glioma stem-like cells [50], and a higher expression of proteins of the pentose phosphate pathway was detected in the brain metastasis of breast cancer [51].

Interestingly, the analysis by the REACTOME tool listed the elevated platelet cytosolic Ca^2+^ and neutrophil degranulation among the most overrepresented pathways. These findings would further enforce the role of inflammation, beta-catenin, innate immunity, calcium, and actin cytoskeleton in the aggressive behavior and pathogenesis of AC, although their interpretation is controversial due to the histological complexity of the tumor tissue and the possible infiltration of immune and inflammatory cells [15]. It is also noteworthy to evidence the overrepresented pathways of vesicle-mediated transport (Table 3) and integrin signaling (Table 2) which could be consistent with the infiltrative behavior and high recurrence rate of AC.

In addition to a thorough review of the most common protein elements characterized in AC samples, we also evaluated the detection and relative distribution of the expression products of genes overexpressed in AC or associated with specific signatures or to potential therapeutic drugs, as reported by previous evidence in the literature [7, 13, 15].

Concerning the recent identification in AC of pharmacological therapy target genes [10], we searched the top 20 protein products reported as overexpressed in AC in comparison to normal brain and other malignancies. According to the sensitivity of the used analytical instrumentation, it is interesting to evidence the identification of MMP12 and MMP9 in 3/7 and 1/7 AC samples, respectively, EpCAM and RRAS in 4/7 and PSMB1 and EGFR in 5/7. In addition, ameloblastin (AMBN) and enamelin (ENAM), two proteins related to odontogenic development and discussed in the same paper as part of the AC gene signature of ectodermal development, were characterized in 5/7 and 2/7 samples together with several keratins related to epidermal development, i.e., KRT5, KRT14, KRT15, and KRT16, in 7/7 samples. Two samples additionally showed the detection of KRT31, KRT34, and KRT85 (the latter found in 3/5 samples) and laminin LAMA3 in 4/7.

MMP9 was also reported to be enclosed in the 16 gene list upregulated in recurrent AC patients [13]. Considering the detection sensitivity of the applied analytical method, no other MMPs, CXCL12, and CXCR4, associated or overexpressed in tumor recurrence [13], were found in the AC solid tissues analyzed. Interestingly, the same sample showing MMP9 also exhibited the detection of cathepsin K (CTSK), integrin beta-3 (ITGB3), and fibronectin (FN1), all enclosed in the 16 genes upregulated in patients with recurrent AC [13]. FN1 was identified in all the seven samples analyzed and ITGB3 in 4 out of 7, while MMP20, associated with the molecular profile of AC and involved in the odontogenesis process, was characterized in only one sample [15]. The stem cell marker CD44, found to be expressed in AC tumor [5], was characterized in 7/7 samples, while CD133 was not detected.

Among the cell adhesion molecules, Fascin-1, upregulated in beta-catenin-accumulating cluster cells and involved in the formation of tumor protrusions [5], was, as well as beta-catenin (CTNNB1), characterized in all AC samples of this study. Moreover, EpCAM (CD326), which showed higher levels in AC compared to papillary craniopharyngioma and RCC [12], was identified in four of them. The tight junction protein claudin-1 was not detected, in accordance with its previously reported reduced expression in AC with respect to other histotypes and RCC [11].

The upregulation of cytokine-encoding genes in whole AC tumor tissue was found to correlate to the presence of an immune infiltrate and the expression of inflammatory cell markers, like CD14 and CD68 [15]. CD14 was identified in 4/7 samples while CD68 was not characterized. According to the limit of detection of the analytical technique used, no cytokines and inflammatory interleukins have been identified, with the exception of CXCL7 present at low level in only one sample.

## 5. Conclusions

Our results are, according to the limit of detection of the analytical technique used, in good agreement with findings reported by previous research in the field and provide a deeper insight on the intact proteome of the AC solid component, enforcing the role of LC-MS proteome profiling for whole tissue molecular characterization.

After a series of studies that highlighted the prominent role of beta-catenin in the upregulation of cellular growth and AC recurrence, this research provides some additional clues to the understanding of the proteins and pathways possibly involved in the process of tumorigenesis typical of this disease. The finding of inflammatory proteins/peptides in the solid component of the AC, in conformity with the results obtained studying the intracystic fluid, could help in establishing the role of tumor cells in cyst formation and growth. The overrepresentation of focal adhesion pathways, including the beta-catenin cascade, may also explain the remarkable proliferative potential and invasiveness of AC, a WHO grade I tumor, and offers further evidence on the connection of beta-catenin to the onset of this pediatric tumor.

The complex composition of AC tumor tissue requires in the future the proteomic analysis of selected cell types. Nonetheless, the present study, first applying a top-down/bottom-up integrated proteomic platform, contributes to outline a preliminary overview of the phenotypic protein asset of the tumor solid component. By defining both the undigested and digested proteomes and their pathway classification, this study provides a glimpse of the still poorly understood complexity that underlies AC tumor pathogenesis and behavior.

## Figures and Tables

**Table 1:** List and distribution of the proteins and peptides identified in AC tissue in at least four out of the seven samples analysed by LC-MS in top-down approach.

Uniprot accession	Description^∗^	Molecular weight^∗∗^ [M+H]^+^	Posttranslational modification (PTM)	Sample distribution^#^						
				AC1	AC2	AC3	AC4	AC5	AC6	AC7
P62328	**Thymosin *β*4** ^**§**^ [18, 23]	**4961.51**	**N-term acetylation**	•	•	•	•	•	•	•
	Thymosin *β*4 oxidized form^**§**^ [23]	4977.51	N-term acetylation; M^6^ oxidation	•	•	•	•	•	—	—
	**Thymosin *β*4 fragment 2-12** ^**§**^ [23]	**1304.61**	**N-term acetylation**	•	•	•	•	•	•	—
	**Thymosin *β*4 fragment 20-44**	**2829.42**		•	•	•	•	•	•	•
	Thymosin *β*4 fragment 20-40	2485.28		•	•	•	—	—	•	—
	Thymosin *β*4 fragment 27-44	1984.99		•	•	—	•	•	•	•
	**Thymosin *β*4 fragment 16-44**	**3285.73**		•	•	•	•	•	•	•
	Thymosin *β*4 fragment 21-44	2701.33		•	—	—	•	•	•	•
	Thymosin *β*4 fragment 20-42	2613.34		•	•	•	—	•	•	—
	Thymosin *β*4 fragment 20-39	2357.23		•	•	•	—	—	•	—
	Thymosin *β*4 fragment 26-44	2113.08		•	•	—	•	•	—	•

P63313	Thymosin *β*10^**§**^ [23]	4934.54	N-term acetylation	•	—	•	•	•	—	•
	Thymosin *β*10 fragment 2-42, *des-IS* ^**§**^ [23]	4734.43	N-term acetylation, C-terminal truncation	•	—	•	•	•	—	—
	Thymosin *β*10 fragment 2-27^**§**^ [23]	2964.51	N-term acetylation	•	—	•	•	•	—	—

P06454-2	**Prothymosin *α* isoform 2** ^**§**^ [23]	**11978.90**	**N-term acetylation**	•	•	•	•	•	•	•

P06454	Prothymosin *α* fragment 2-29,*Thymosin α1* ^**§**^ [23]	3107.52	N-term acetylation	•	—	•	•	•	—	—
	Prothymosin *α* fragment 2-36,*Thymosin α11* ^**§**^ [23]	3788.83	N-term acetylation	•	•	•	•	•	•	—
	Prothymosin *α* fragment 90-111	2553.08		•	•	—	•	•	•	—

P59665	**Alpha-defensin1** ^**§**^ [16, 17]	**3440.53**	**Disulfide bonds C** ^**2**^ **-C** ^**30**^ **; C** ^**4**^ **-C** ^**19**^ **; C** ^**9**^ **-C** ^**29**^	•	•	•	•	•	•	•

P59665	**Alpha-defensin2** ^**§**^ [16, 17]	**3369.50**	**Disulfide bonds C** ^**1**^ **-C** ^**29**^ **; C** ^**3**^ **-C** ^**18**^ **; C** ^**8**^ **-C** ^**28**^	•	•	•	•	•	•	•

P59666	**Alpha-defensin3** ^**§**^ [16, 17]	**3484.52**	**Disulfide bonds C** ^**2**^ **-C** ^**30**^ **; C** ^**4**^ **-C** ^**19**^ **; C** ^**9**^ **-C** ^**29**^	•	•	—	•	•	•	•

P12838	Alpha-defensin4^**§**^ [16, 17]	3707.77	Disulfide bonds C^2^-C^30^; C^4^-C^19^; C^9^-C^29^	—	•	•	•	—	•	—

P02671	**Fibrinogen alpha chain fragment 20-35, *fibrinopeptide A***	**1536.69**		•	•	•	•	•	•	•

P14136	Glial fibrillary acidic protein fragment 388-432	5206.73		•	•	—	•	—	—	•
	Glial fibrillary acidic protein fragment 398-432	4035.12		•	•	—	•	•	—	•
	Glial fibrillary acidic protein fragment 406-432	3197.67		•	•	—	•	—	—	•
	Glial fibrillary acidic protein fragment 416-430	1797.91		•	•	—	•	•	—	•
	Glial fibrillary acidic protein fragment 414-432	2288.11		•	•	—	•	•	—	•
	**Glial fibrillary acidic protein fragment 406-429**	**2852.55**		•	•	•	•	•	•	•
	Glial fibrillary acidic protein fragment 389-405	1900.01		•	•	—	•	•	—	—
	Glial fibrillary acidic protein fragment 388-405	2028.08		•	•	—	•	•	—	•
	Glial fibrillary acidic protein fragment418-432	1756.89		•	•	—	•	•	—	•
	Glial fibrillary acidic protein fragment 414-430	2058.00		•	•	—	•	•	—	•
	**Glial fibrillary acidic protein fragment 416-432**	**2028.02**		•	•	—	•	•	•	•
	Glial fibrillary acidic protein fragment 412-432	2488.23		•	•	—	•	•	—	•
	Glial fibrillary acidic protein fragment 384-397	1651.86		•	•	—	•	—	—	•
	Glial fibrillary acidic protein fragment 389-398	1149.61		•	•	—	•	•	—	•
	Glial fibrillary acidic protein fragment 412-431	2357.23		•	•	—	•	•	—	—
	Glial fibrillary acidic protein fragment 401-432	3761.98		•	•	—	•	•	—	—
	Glial fibrillary acidic protein fragment 407-432	3041.58		•	•	—	•	—	—	•
	Glial fibrillary acidic protein fragment 413-432	2387.17		•	•	—	•	—	—	•
	Glial fibrillary acidic protein fragment 412-430	2258.11		•	•	—	•	•	—	—
	Glial fibrillary acidic protein fragment 415-432	2159.06		•	—	—	•	•	—	•
	Glial fibrillary acidic protein fragment 415-430	1928.95		•	•	—	•	•	—	—
	Glial fibrillary acidic protein fragment 417-432	1871.92		•	•	—	•	•	—	•
	Glial fibrillary acidic protein fragment 419-432	1699.87		•	•	—	•	•	—	—
	Glial fibrillary acidic protein fragment 416-429	1682.88		•	•	—	•	—	—	•
	Glial fibrillary acidic protein fragment 416-428	1554.79		•	•	—	•	•	—	•
	Glial fibrillary acidic protein fragment 418-430	1526.78		•	•	—	•	•	—	•
	Glial fibrillary acidic protein fragment 385-397	1504.80		•	•	—	•	•	—	—
	Glial fibrillary acidic protein fragment 389-401	1464.76		•	•	—	•	•	—	—
	Glial fibrillary acidic protein fragment 386-397	1417.77		•	•	—	•	—	—	•

P08670	Vimentin fragment 434-466	3814.94		—	•	•	•	—	•	—
	Vimentin fragment 445-466	2551.21		•	•	•	•	—	•	—
	**Vimentin fragment 444-466**	**2664.29**		•	•	•	•	•	•	•
	**Vimentin fragment 443-466**	**2777.38**		•	•	•	•	•	•	•
	**Vimentin fragment 446-466**	**2423.11**		•	•	•	•	•	•	—
	**Vimentin fragment 450-466**	**1992.90**		•	•	•	•	•	•	•
	Vimentin fragment 13-28 or 12-27	1650.82		—	•	•	•	—	•	—
	Vimentin fragment 440-466	3147.60		•	•	—	•	—	•	—
	Vimentin fragment 447-466	2322.06		•	•	•	•	—	•	—
	**Vimentin fragment 441-466**	**2991.51**		•	•	•	•	•	•	—
	Vimentin fragment 442-466	2890.46		•	•	—	•	—	•	—
	Vimentin fragment 5-28	2504.21		•	•	•	•	—	—	—
	Vimentin fragment 29-50	2391.23		•	•	•	•	—	—	—
	Vimentin fragment 424-442	2195.19		•	•	—	•	—	•	—
	Vimentin fragment 451-466	1836.80		•	•	—	•	—	•	—
	Vimentin fragment 452-466	1721.77		—	•	—	•	•	•	—
	Vimentin fragment 453-466	1664.76		•	•	—	•	•	—	—
	Vimentin fragment 454-466	1536.69		•	•	—	•	•	•	—

P0CG47	**Ubiquitin** ^§^ [14]	**8560.64**		•	•	•	•	•	•	•
	**Ubiquitin fragment 1-74, *des-GG*** ^§^ [23]	**8446.60**	**C-terminal truncation**	•	•	•	•	•	•	—
	Ubiquitin fragment 1-73, *des-RGG* ^§^ [23]	8290.51	C-terminal truncation	—	•	•	—	•	•	—

P06703	**Protein S100-A6 cysteinylated form** ^§^ [17, 52]	**10204.33**	**N-term acetylation; cysteinylation**	•	•	•	•	—	•	•

P31949	Protein S100-A11^§^ [52, 53]	11644.79	N-term acetylation	•	—	•	•	—	•	—

P69905	**Hemoglobin subunit alpha fragment 2-34**	**3473.77**		•	•	•	•	•	•	•
	Hemoglobin subunit alpha fragment 2-33	3326.70		—	•	•	•	—	•	—
	**Hemoglobin subunit alpha fragment 111-142**	**3427.83**		•	•	•	•	•	•	—
	Hemoglobin subunit alpha fragment 107-142	3854.12		•	•	—	•	—	•	—
	Hemoglobin subunit alpha fragment 110-142	3540.91		—	•	•	•	—	•	—
	Hemoglobin subunit alpha fragment 16-34	1992.97		•	•	—	•	—	•	—
	Hemoglobin subunit alpha fragment 34-47	1732.87		—	—	•	•	•	•	—

P68871	Hemoglobin subunit beta fragment 131-147	1869.01		—	—	•	•	•	•	—
	Hemoglobin subunit beta fragment 33-42, *LVV-hemorphin-7*	1308.71		•	•	•	•	—	•	—

P0DP23	**Calmodulin** ^§^ [52]	**16780.91**	**2 acetylations (or 1 acetylation and 1 trimethylation)**	•	•	•	•	•	•	•

P01011	**Alpha-1-antichymotrypsin fragment 387-423**	**4352.34**	**C-terminal fragment**	•	•	•	•	•	•	•
	Alpha-1-antichymotrypsin fragment 390-423	4023.19	C-terminal fragment	•	•	•	•	—	—	•
	Alpha-1-antichymotrypsin fragment 384-423	4623.52	C-terminal fragment	—	•	—	•	—	•	•

P01009	Alpha-1-antitrypsin fragment 383-418	4133.24	C-terminal fragment	—	•	—	•	—	•	•
	**Alpha-1-antitrypsin fragment 384-418**	**4046.20**	**C-terminal fragment**	•	•	•	•	—	•	•

P80723	Brain acid-soluble protein 1 fragment 199-227	2892.43	C-terminal fragment	—	—	•	—	•	•	•

P55087	Aquaporin-4 fragment 275-303	3257.61		•	•	—	•	•	—	•

P02768	Serum albumin fragment 25-48	2753.44		—	•	•	•	—	•	•

P16403; P16402; P10412	Histone H1.2 fragment 33-53/histone H1.3 fragment 34-54/histone H1.4 fragment 33-53	2139.21		•	•	•	•	—	•	—

P05204	Non-histone chromosomal protein HMG-17 fragment 29-42	1471.86		•	—	•	•	—	•	—

O60814	Histone H2B type 1-K fragment 2-12	1092.61		•	•	—	•	•	•	—
	Histone H2B type 1-K fragment 2-16	1492.85		•	•	—	•	•	—	—

Q5QNW6	Histone H2B type 2-F fragment 2-17	1606.93		•	•	—	•	•	—	—

P17677	Neuromodulin fragment 220-238	2154.92		•	•	—	•	•	—	—

P46821	Microtubule-associated protein 1B fragment 853-874	2624.36		•	•	—	•	•	—	—

P68363	Tubulin alpha-1B fragment 68-79	1384.73		•	•	—	•	•	—	—

^§^Based on previous identification, relative reference reported. ^∗^In bold are marked the protein/peptides identified in at least six of the seven samples analyzed. ^∗∗^Monoisotopic. ^#^The “•” and “-” symbols indicate detected and undetected proteins/peptides, respectively.

**Table 2:** List of the pathways resulting from PANTHER database pathway overrepresentation analysis.

PANTHER pathways	Homo sapiens—REFLIST (21042)	Client text box input (436)^∗^	Client text box input (expected)	Client text box input (over/under)	Client text box input (fold enrichment)	Client text box input (raw *P* value)	Client text box input (FDR)
Pentose phosphate pathway (P02762)	8	5	0.17	+	30.16	3.79*E* − 06	8.83*E* − 05
Glycolysis (P00024)	20	10	0.41	+	24.13	2.24*E* − 10	1.83*E* − 08
ATP synthesis (P02721)	7	3	0.15	+	20.68	8.96*E* − 04	1.62*E* − 02
TCA cycle (P00051)	10	3	0.21	+	14.48	2.04*E* − 03	2.56*E* − 02
Plasminogen-activating cascade (P00050)	18	4	0.37	+	10.72	9.17*E* − 04	1.49*E* − 02
Blood coagulation (P00011)	46	9	0.95	+	9.44	1.50*E* − 06	4.07*E* − 05
Parkinson disease (P00049)	103	16	2.13	+	7.5	2.52*E* − 09	1.37*E* − 07
FAS signaling pathway (P00020)	34	5	0.7	+	7.1	1.10*E* − 03	1.63*E* − 02
Cytoskeletal regulation by Rho GTPase (P00016)	84	12	1.74	+	6.89	5.64*E* − 07	1.84*E* − 05
Huntington disease (P00029)	145	17	3	+	5.66	3.59*E* − 08	1.46*E* − 06
Dopamine receptor-mediated signaling pathway (P05912)	58	6	1.2	+	4.99	1.88*E* − 03	2.55*E* − 02
Integrin signalling pathway (P00034)	190	14	3.94	+	3.56	7.78*E* − 05	1.59*E* − 03
Unclassified (UNCLASSIFIED)	18438	311	382.04	—	0.81	3.91*E* − 19	6.38*E* − 17

^∗^Referred to the experimental protein UniProt accession input list on PANTHER analysis tool.

**Table 3:** Top 25 pathways' list resulting from REACTOME database pathway overrepresentation analysis.

Pathway name	# entities found	# entities total	Entity ratio	Entity *P* value	Entity FDR	# reactions found	# reactions total	Reaction ratio
Neutrophil degranulation	84	480	0.034	1.11*E* − 16	2.32*E* − 14	10	10	8.48*E* − 04
Platelet degranulation	40	137	0.010	1.11*E* − 16	2.32*E* − 14	6	11	9.33*E* − 04
Platelet activation, signaling, and aggregation	47	293	0.021	1.11*E* − 16	2.32*E* − 14	53	114	9.67*E* − 03
Response to elevated platelet cytosolic Ca^2+^	40	144	0.010	1.11*E* − 16	2.32*E* − 14	6	14	1.19*E* − 03
Innate immune system	134	1302	0.093	1.11*E* − 16	2.32*E* − 14	243	651	5.52*E* − 02
Immune system	170	2641	0.189	1.11*E* − 16	2.32*E* − 14	415	1493	1.27*E* − 01
Hemostasis	75	812	0.058	3.33*E* − 16	5.96*E* − 14	129	327	2.77*E* − 02
Regulation of insulin-like growth factor (IGF) transport and uptake by insulin-like growth factor-binding proteins (IGFBPs)	27	127	0.009	3.63*E* − 14	5.66*E* − 12	3	14	1.19*E* − 03
Posttranslational protein phosphorylation	25	109	0.008	6.28*E* − 14	8.73*E* − 12	1	1	8.48*E* − 05
Vesicle-mediated transport	63	820	0.059	2.51*E* − 10	3.13*E* − 08	160	251	2.13*E* − 02
Cellular responses to stress	46	512	0.037	7.73*E* − 10	8.16*E* − 08	67	184	1.56*E* − 02
Detoxification of reactive oxygen species	16	65	0.005	7.85*E* − 10	8.16*E* − 08	21	34	2.88*E* − 03
Regulation of complement cascade	22	139	0.010	2.01*E* − 09	1.93*E* − 07	40	42	3.56*E* − 03
Metabolism of proteins	125	2342	0.167	4.52*E* − 09	3.87*E* − 07	269	883	7.49*E* − 02
Gene and protein expression by JAK-STAT signaling after interleukin-12 stimulation	16	74	0.005	4.80*E* − 09	3.87*E* − 07	16	36	3.05*E* − 03
Interleukin-12 signaling	17	85	0.006	4.96*E* − 09	3.87*E* − 07	18	56	4.75*E* − 03
Interleukin-12 family signaling	18	97	0.007	5.51*E* − 09	4.02*E* − 07	24	114	9.67*E* − 03
Smooth muscle contraction	12	39	0.003	9.38*E* − 09	6.47*E* − 07	8	9	7.63*E* − 04
Apoptosis	24	180	0.013	9.84*E* − 09	6.49*E* − 07	29	126	1.07*E* − 02
Complement cascade	22	156	0.011	1.54*E* − 08	9.57*E* − 07	62	71	6.02*E* − 03
Programmed cell death	24	188	0.013	2.21*E* − 08	1.30*E* − 06	30	139	1.18*E* − 02
Cellular responses to external stimuli	47	599	0.043	3.16*E* − 08	1.80*E* − 06	68	254	2.15*E* − 02
Apoptotic execution phase	13	54	0.004	3.92*E* − 08	2.12*E* − 06	19	57	4.84*E* − 03
Axon guidance	44	583	0.042	2.58*E* − 07	1.34*E* − 05	52	297	2.52*E* − 02

## Data Availability

The data used to support the findings of this study are included within the article.

## Supplementary Materials

Supplementary Materials The supplementary data file encloses the top-down identification data from Proteome Discoverer software elaboration and manual identifications (S1 and S2, respectively), the Proteome Discoverer software multireport data file of bottom-up identifications (S3), the input gene list for pathway classification analysis, the PANTHER pathway classification results and their relative gene component lists (S4, S5, and S6, respectively), and the PANTHER and REACTOME pathway overrepresentation analysis results (S7 and S8, respectively). Supplementary Table S1 reports the distribution of the identified protein elements within the AC samples analysed.
